# Androgen-Regulated Transcriptional Control of Sialyltransferases in Prostate Cancer Cells

**DOI:** 10.1371/journal.pone.0031234

**Published:** 2012-02-08

**Authors:** Koji Hatano, Yasuhide Miyamoto, Masaki Mori, Keisuke Nimura, Yasutomo Nakai, Norio Nonomura, Yasufumi Kaneda

**Affiliations:** 1 Division of Gene Therapy Science, Graduate School of Medicine, Osaka University, Osaka, Japan; 2 Department of Urology, Graduate School of Medicine, Osaka University, Osaka, Japan; 3 Department of Immunology, Osaka Medical Center for Cancer and Cardiovascular Diseases, Osaka, Japan; Florida International University, United States of America

## Abstract

The expression of gangliosides is often associated with cancer progression. Sialyltransferases have received much attention in terms of their relationship with cancer because they modulate the expression of gangliosides. We previously demonstrated that GD1a production was high in castration-resistant prostate cancer cell lines, PC3 and DU145, mainly due to their high expression of β-galactoside α2,3-sialyltransferase (ST3Gal) II (not ST3Gal I), and the expression of both ST3Gals was regulated by NF-κB, mainly by RelB. We herein demonstrate that GD1a was produced in abundance in cancerous tissue samples from human patients with hormone-sensitive prostate cancers as well as castration-resistant prostate cancers. The expression of ST3Gal II was constitutively activated in castration-resistant prostate cancer cell lines, PC3 and DU145, because of the hypomethylation of CpG island in its promoter. However, in androgen-depleted LNCap cells, a hormone-sensitive prostate cancer cell line, the expression of ST3Gal II was silenced because of the hypermethylation of the promoter region. The expression of ST3Gal II in LNCap cells increased with testosterone treatment because of the demethylation of the CpG sites. This testosterone-dependent ST3Gal II expression was suppressed by RelB siRNA, indicating that RelB activated ST3Gal II transcription in the testosterone-induced demethylated promoter. Therefore, in hormone-sensitive prostate cancers, the production of GD1a may be regulated by androgen. This is the first report indicating that the expression of a sialyltransferase is transcriptionally regulated by androgen-dependent demethylation of the CpG sites in its gene promoter.

## Introduction

Many cancer cells have aberrant sialylated glycans on their surface. These aberrant molecules may be involved in cancer progression [1]–[3], but sialylated glycans also play many roles in healthy organisms and non-cancer cells, including embryogenesis, regulation of the immune response and virus binding that leads to infections [4], [5]. Sialylated glycans are synthesized by sialyltransferases, which add sialic acids to the oligosaccharide chains of glycoproteins and glycosphingolipids (GSLs) [5]. To date, 20 sialyltransferase genes have been cloned, and the respective enzymes have been grouped into four families according to the carbohydrate linkages they catalyze: β-galactoside α2,3-sialyltransferases (ST3Gal I–VI), β-galactoside α2,6-sialyltransferases (ST6Gal I and II), GalNAc α2,6-sialyltransferases (ST6GalNAc I–VI), and α2,8-sialyltransferases (ST8Sia I–VI) [6]. During neoplastic transformation and cancer progression, the activity of sialyltransferases is often altered, and consequently, cancer cells have more heavily sialylated glycans on their surface than non-cancer cells [1], [2], [7].

GSLs that contain sialic acids are known as gangliosides and are expressed at high levels in various cancer cells [3]. The gangliosides present on cancer cells are used as biomarkers or treatment targets, and the enriched gangliosides differ between cancer cell types [8]–[10]. We have focused on GD1a synthesis in cancer cells because GD1a has several biological actions that promote cancer progression. For example, highly metastatic cancer cells have abundant GD1a, and GD1a is involved in cancer cell adhesion to endothelial cells during metastasis [11]. The GD1a shed by tumor cells in the tumor microenvironment promotes angiogenesis and enhances growth factor signaling by increasing the dimerization of growth factor receptors [12]–[15]. Therefore, GD1a may be involved in cancer cell proliferation and metastasis. Furthermore, this ganglioside is a receptor for the Sendai virus [16], and inactivated Sendai virus particles [hemagglutinating virus of Japan envelope (HVJ-E)] induce apoptosis in several human cancer cells with enriched GD1a on their surface [17]. Therefore, GD1a may be an attractive molecule from the viewpoint of cancer therapy.

GD1a has been reported to be abundantly produced in castration-resistant prostate cancer cells [17]–[20], and we previously demonstrated that castration-resistant prostate cancer cells were effectively eradicated by HVJ-E [17]. GD1a is synthesized from GM1 by ST3Gal I and II. The Km value of ST3Gal II for GM1 is smaller than that of ST3Gal I; thus, ST3Gal II preferentially contributes to GD1a synthesis [6], [21]–[24]. We recently demonstrated that abundant production of GD1a in castration-resistant prostate cancer cells is correlated with the high levels of ST3Gal II expression [20] and that ST3Gal II expression is regulated by NF-κB, mainly by RelB, in castration-resistant prostate cancer cells [20]. Although the RelB levels were similar in a hormone-sensitive prostate cancer cell line (LNCap) and castration-resistant prostate cancer cells, and although ST3Gal I was expressed in LNCap cells [20], the expression of ST3Gal II was silenced in LNCap cells, and GD1a was much less abundant in the LNCap cells [17], [20].

There has so far been no published analysis of the ganglioside levels in cancerous tissue samples from human patients with prostate cancer; however, an endogenous immune response to GD1a was observed in patients with hormone-sensitive prostate cancer, but not in healthy controls [19], thus suggesting that GD1a is abundantly produced in hormone-sensitive prostate cancers. Prostate cancer exhibits androgen-dependent growth and progression [25]; therefore, androgens may also regulate the GD1a production that is related to cancer progression. However, there have also been no published studies that have examined the hormonal control of sialylated glycan synthesis.

The aim of this study was to determine whether GD1a is produced in abundance in hormone-sensitive prostate cancers in patients and to analyze the transcriptional control of sialyltransferases, especially ST3Gal II, required for the synthesis of GD1a in hormone-sensitive prostate cancers.

## Materials and Methods

### Ethics statement

Written informed consent was obtained from all patients for the use of their tissue specimens, and the use of such specimens was approved by the Osaka University Hospital Institutional Review Board (Osaka, Japan).

### Cell culture

Castration-resistant human prostate cancer cell lines, PC3 and DU145, and a hormone-sensitive human prostate cancer cell line, LNCap clone FGC, were purchased from the American Type Culture Collection (Rockville, MD). A normal human prostatic epithelial cell, PNT2, was purchased from the European Collection of Animal Cell Cultures (Porton Down, UK). PC3 cells were maintained in Dulbecco's modified Eagle F12 medium (Nacalai Tesque, Kyoto, Japan), and DU145, LNCap, and PNT2 cells were maintained in RPMI 1640 medium (Nacalai Tesque, Kyoto, Japan). All media were supplemented with 10% fetal bovine serum (FBS), 100 U/ml penicillin, and 100 µg/ml streptomycin. The cells were incubated at 37°C in a humidified atmosphere of 95% air and 5% CO_2_.

### Reagents and antibodies

Trichostatin A (TSA) and 5-aza-2′-deoxycytidine (5-azadC) were purchased from Wako Pure Chemical Industries (Osaka, Japan). Testosterone was purchased from Tokyo Chemical Industry (Tokyo, Japan). Bicalutamide was purchased from Enzo Life Sciences (Plymouth Meeting, PA). Restriction enzymes, *Msp*I and *Hpa*II, were purchased from New England Biolabs (Ipswich, MA). Anti-human RelB (C1E4) was purchased from Cell Signaling (Danvers, MA). Anti-human β-actin (AC-15) was purchased from Abcam (Cambridge, UK).

### Real-time quantitative RT-PCR

Total RNA was isolated using an RNeasy RNA isolation kit (Qiagen, Valencia, CA). The cDNA was synthesized using a High Capacity cDNA Reverse Transcription Kit (Applied Biosystems, Foster City, CA). Real-time quantitative PCR was performed with an Applied Biosystems 7900 HT Fast Real-Time PCR system under the following conditions: 95°C for 10 min followed by 40 cycles of 95°C for 15 s and 60°C for 1 min. Mixtures of probes and primer pairs specific for human ST3Gal I (Hs00161688_m1), ST3Gal II (Hs00199480_m1), ST3Gal VI (Hs00196086_m1), RelB (Hs00232399_m1), and glyceraldehyde-3-phosphate dehydrogenase (GAPDH) (Hs99999905_m1) were purchased from Applied Biosystems. The relative expression levels were calculated from a standard curve obtained using log dilutions of cDNA containing the gene of interest, and values were normalized to GAPDH, an internal control.

### Evaluation using a reporter gene

Genes were transfected into cells along with a luciferase reporter construct driven by a NF-κB binding site, RelA, and RelB (NF-κB luciferase reporter gene; BD Bioscience Clontech, Palo Alto, CA), using the Fugene HD reagent (Roche, Basel, Switzerland). The luciferase activity was measured with the dual-luciferase assay system (Promega, Madison, WI).

### Western blot analysis

The cells were harvested and lysed with RIPA lysis buffer. Protein samples were separated by sodium dodecyl sulfate polyacrylamide gel electrophoresis. The separated proteins were transferred onto polyvinylidene fluoride membranes, then the membranes were blocked with 5% skim milk and incubated overnight at 4°C with anti-RelB (1∶500) or anti-β-actin (1∶2000) antibodies. The membranes were washed and labeled with a 1∶2000 dilution of horseradish peroxidase–conjugated secondary antibody (GE Healthcare, Buckinghamshire, UK) at room temperature for approximately 1 hour. Detection by chemiluminescence was performed according to the ECL user's guide (Amersham, Buckinghamshire, UK). Images were captured with ImageQuant LAS 4000mini (GE Healthcare, Buckinghamshire, UK), and quantification of Western blot signals was performed by densitometry with ImageQuant TL software (GE Healthcare, Buckinghamshire, UK).

### RNA interference experiments

The following double-strand stealth small interfering RNA (siRNA) oligonucleotides and scrambled RNA were purchased from Invitrogen (Tokyo, Japan): siRNA oligonucleotides against RelB were (sense) 5′-UCUUCAGGGACCCAGCGUUGUAGGG-3′ and (antisense) 5′-CCCUACAACGCUGGGUCCCUGAAGA-3′. Transfections were performed with lipofectamine RNAiMAX (Invitrogen, Tokyo, Japan), according to the manufacturer's instructions.

### Methylation-specific PCR (MSP) analysis

DNA methylation was examined at the CpG islands by a MSP analysis as previously reported [26]. For the MSP analysis, genomic DNA was extracted from cells and purified using the QIAamp DNA kit (Qiagen, Valencia, CA). Genomic DNA was subjected to bisulfite conversion using an EZ DNA Methylation Kit (Zymo Research, Irvine, CA). Based on the sequence of the ST3Gal II p1 promoter and ST3Gal I p1 promoter, methylated-specific primers and unmethylated-specific primers were designed using the Methyl Primer Express Software program version 1.0 (Applied Biosystems, Foster City, CA). The ST3Gal II-methylated-specific primers were sense, 5′-TAGGGCGTAGCGGTTTTATC-3′, antisense, 5′-ACTAACCGAAAACGCCTCTC-3′, and the ST3Gal II-unmethylated-specific primers were sense, 5′-GGTTAGGGTGTAGTGGTTTTATT-3′, and antisense, 5′-CACACTAACCAAAAACACCTCTC-3′. The ST3Gal II 5′-untranslated region from −659 to −495 was chosen for the MSP analysis. The ST3Gal I-methylated-specific primers were sense, 5′-TAGGGTCGGTCGTAGTGTTC-3′, antisense, 5′-ACCGATCCCCTACTAACGAC-3′, and the ST3Gal I-unmethylated-specific primers were sense, 5′-TTAGGGTTGGTTGTAGTGTTT-3′, and antisense, 5′-AACCAATCCCCTACTAACAAC-3′. The ST3Gal I 5′-untranslated region from −697 to −535 was chosen for the MSP analysis. The glutathione S-transferase-π gene (GSTP1) -methylated-specific primers were sense, 5′-AGTTGCGCGGCGATTTC-3′, antisense, 5′-GCCCCAATACTAAATCACGACG-3′, and the GSTP1-unmethylated-specific primers were sense, 5′-GATGTTTGGGGTGTAGTGGTTGTT-3′, and antisense, 5′-CCACCCCAATACTAAATCACAACA-3′, as described previously [27]. Purified genomic DNA treated with sodium bisulfite was amplified by PCR as follows: 2 min at 95°C for denaturation, 35 cycles of amplification (95°C for 30 s, 56°C for 30 s, and 72°C for 30 s). Human genomic DNA or enzymatically methylated human genomic DNA (Chemicon International, Temecula, CA) was bisulfite-converted and used as a positive control for the unmethylated or methylated genes. The absence of a DNA template served as a negative control. The products were analyzed in 2% agarose gels stained with ethidium bromide.

### Isolation of acidic GSLs from prostate cancer tissues

Patients diagnosed with prostate cancer had undergone prostate biopsy or resection of tumors at Osaka University Hospital (Osaka, Japan). Primary cancerous tissue samples were frozen in liquid nitrogen and stored at −80°C until use. The majority of the experimental procedures have been reported previously [28]. In brief, the samples were homogenized in chloroform/methanol (2∶1, v/v), and incubated at room temperature for 2 h with 30 s of sonication every 30 min. Methanol was then added to the samples, which were centrifuged at 1800×g for 15 min. The pellets were homogenized in chloroform/methanol/water (1∶2∶0.8, v/v/v), incubated at room temperature for 2 h, and then centrifuged at 1800×g for 15 min. Both extracts were combined and evaporated to dryness in a vacuum concentrator. The residue was dissolved in chloroform/methanol/water (30∶60∶8) and fractionated by DEAE–Sephadex A25 column chromatography to separate neutral GSLs from acidic GSLs.

### Analysis of acidic GSLs

The structures of the acidic GSLs were analyzed by enzymatic release of carbohydrate moieties, fluorescent labeling with aminopyridine, and two-dimensional mapping followed by mass spectrometry. The majority of experimental procedures have been reported previously [28]. In brief, the acidic GSLs were extracted from primary cancer tissue samples or cultured cancer cells and digested with recombinant endoglycoceramidase II from *Rhodococcus* sp. (Takara Bio Inc., Shiga, Japan). The released oligosaccharides were labeled with 2-aminopyridine (2-AP) and separated on a Shimadzu LC-20A HPLC system (Shimadzu Corporation, Kyoto, Japan) equipped with a Waters 2475 fluorescence detector. Normal-phase HPLC was performed on a TSK gel Amide-80 column (0.2×25 cm, Tosoh, Tokyo, Japan). The molecular size of each pyridylaminated (PA)-oligosaccharide is given in glucose units (Gu) based on the elution times of PA-isomaltooligosaccharides. Reversed-phase HPLC was performed on a TSK gel ODS-80Ts column (0.2×15 cm, Tosoh). The retention time of each PA-oligosaccharide is given in glucose units based on the elution times of the PA-isomaltooligosaccharides. Therefore, the behaviors of a given compound in these two columns provide a unique set of Gu (amide) and Gu (ODS) values, which correspond to coordinates on a 2-D map. PA-oligosaccharides were analyzed by LC/ESI MS/MS. Standard PA-oligosaccharides, PA-GM1 and PA-GD1a, were purchased from Takara Bio, and PA-LST-a and PA-SPG were isolated as in our previous study [28].

### Statistical analyses

The results are reported as the means ± standard error (S.E.). The two-tailed unpaired Student's *t*-test was used to determine the statistical significance of the differences between two groups. Probability values of P<0.05 were considered to be statistically significant. The statistical analysis was performed using the StatView 5.0 software program (SAS Institute, Cary, NC).

## Results

### Analyses of gangliosides in cancerous tissue samples from patients with prostate cancer

**Figure 1:**
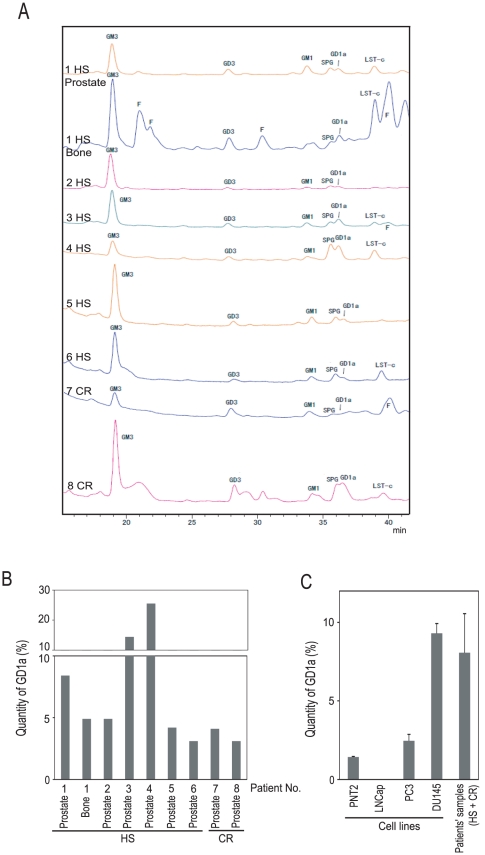
The results of the analyses of gangliosides in cancerous tissue samples from human prostate cancer patients. (A) The acidic GSLs from the cancerous tissue samples from eight patients with prostate cancer, including six patients with advanced hormone-sensitive prostate cancer and two patients with castration-resistant prostate cancer were separated by the molecular size of the oligosaccharides using normal-phase HPLC. Samples from one patient (designated Case 1) were taken from both the prostate and bone metastases for evaluation. (B) The acidic GSLs in the primary cancerous tissue samples were separated by the molecular size of the oligosaccharides using HPLC. The quantity of GD1a is presented as a percentage of the total acidic GSLs with GD1a. (C) The acidic GSLs in cultured prostate cancer cells were separated by the molecular size of the oligosaccharides using HPLC. The assay was done in triplicate, and the means ± S.E. GD1a levels are shown as the ratio to the total acidic GSLs in the cell lines. The mean ± S.E. GD1a level was also presented as the ratio to the total acidic GSLs in the patients' samples (HS+CR) indicated in Figure 1B. (HS, hormone-sensitive; CR, castration-resistant; F, free glycan).

We previously demonstrated that GD1a was abundant in castration-resistant prostate cancer cell lines (including PC3 and DU145), while it was barely detectable in a hormone-sensitive prostate cancer cell line (LNCap) and a normal prostate epithelial cell line (PNT2) [17]. We examined the levels of gangliosides in samples of cancerous tissue from eight patients with prostate cancer, including six patients with advanced hormone-sensitive prostate cancer and two patients with castration-resistant prostate cancer (Table 1). The acidic GSLs extracted from cancerous tissue samples from these patients were examined using HPLC (Fig. 1). Both GM3 and GD3 are common gangliosides expressed in both prostate cancer cells and normal prostate epithelial cells [18], [19]. GD1a was produced in the cancerous tissue samples from both the patients with hormone-sensitive prostate cancers and those with castration-resistant prostate cancers (Fig. 1A, 1B). In all of the patient' samples (hormone-sensitive and castration-resistant), the mean percentage of total acidic GSLs with GD1a was 8.1%, and no statistically significant difference was seen compared with the value from castration-resistant prostate cancer cell lines (PC3 and DU145) (Fig. 1C).

**Table 1:** Patient characteristics.

Patient	Site	HS/CR	PSA	Gleason sum
1	Prostate/Bone metastasis	HS	706	8
2	Prostate	HS	914	9
3	Prostate	HS	2800	9
4	Prostate	HS	3.1	9
5	Prostate	HS	639	9
6	Prostate	HS	2296	9
7	Prostate	CR	36	-
8	Prostate	CR	6.2	-

HS, Hormone-sensitive; CR, Castration-resistant.

### Androgen-dependent regulation of ST3Gal II in LNCap cells

The synthesis of GD1a is mainly regulated by ST3Gal II, and the expression of ST3Gal II is regulated by NF-κB, mainly by RelB, in castration-resistant prostate cancer cell lines [20]. The amounts of nuclear RelB were similar in hormone-sensitive LNCap cells and castration-resistant PC3 and DU145 cells [20], but the expression of ST3Gal II was lower in the LNCap cells than in the PC3 and DU145 cells [20].

**Figure 2:**
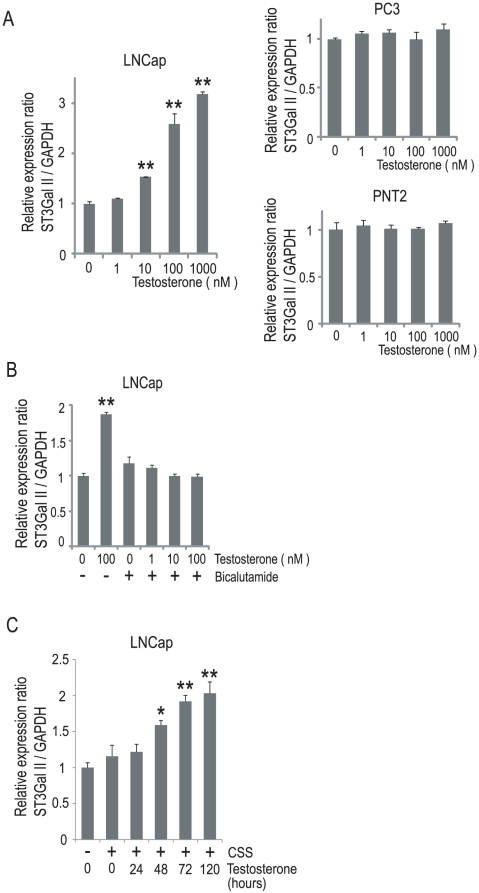
Androgen-dependent regulation of ST3Gal II in LNCap cells. (A) LNCap, PC3, and PNT2 cells were treated with or without testosterone (0–1000 nM) for 120 h, by refeeding with fresh medium with or without testosterone at 72 h. The quantitative real-time PCR analyses of ST3Gal II mRNA were performed, and the expression levels are reported as the means ± S.E. (n = 3) of the fold difference in mRNA after normalizing the values to the expression level of untreated cells. **P<0.001. (B) LNCap cells were treated with or without testosterone (0–100 nM) and simultaneously with or without 10 µM bicalutamide for 120 h, by refeeding with fresh medium with or without testosterone and/or bicalutamide at 72 h. The quantitative real-time PCR analyses for ST3Gal II were performed, and the expression levels are reported as the means ± S.E. (n = 3) of the fold difference in mRNA after normalizing the values to the expression level of untreated cells. **P<0.001. (C) LNCap cells were incubated in charcoal-stripped serum (CSS) for 48 h and then treated with 100 nM testosterone for the indicated times. The quantitative real-time PCR analyses for ST3Gal II were performed, and the expression levels are reported as the means ± S.E. (n = 3) of the fold difference in mRNA after normalizing the values to the expression level of untreated cells. *P<0.05, **P<0.001.

The LNCap cell culture medium is routinely supplemented with 10% fetal bovine serum (FBS). A recent report showed that media supplemented with 10% FBS contains only castrate levels of testosterone [29]; in contrast, hormone-sensitive prostate cancers of untreated patients usually grow in an environment containing testosterone *in vivo*. To analyze the transcriptional control of ST3Gal II in hormone-sensitive prostate cancers, we examined whether the expression of ST3Gal II was controlled by testosterone in LNCap cells. LNCap cells were treated with testosterone (0–1000 nM), and were incubated for 120 h. The quantitative real-time PCR analyses showed that the expression of ST3Gal II was higher in LNCap cells treated with testosterone than in the LNCap cells that were not (Fig. 2A). Furthermore, the induction of ST3Gal II after testosterone treatment was suppressed by an anti-androgen, bicalutamide, in LNCap cells (Fig. 2B). To ensure that there were no androgens present in the media, LNCap cells were incubated in charcoal-stripped serum for 48 h. The basal level of ST3Gal II was not significantly different between the 10% FBS- and charcoal stripped serum-supplemented LNCap cells (Fig. 2C). The LNCap cells were subsequently treated with 100 nM testosterone, and the time-course of expression following testosterone treatment was evaluated. The expression of ST3Gal II was increased 48 h after testosterone treatment, and remained elevated for more than 120 h in the LNCap cells (Fig. 2C). To evaluate the NF-κB activity after testosterone treatment, LNCap cells were transfected with an NF-κB luciferase reporter construct and incubated for 120 h with or without testosterone. The NF-κB activity was not significantly different in the testosterone-treated LNCap cells compared to the cells cultured without testosterone (Figure S1). In PC3 and PNT2 cells, no significant increase in the expression of ST3Gal II was detected regardless of whether the cells cultured with or without testosterone (Fig. 2A). The expression of ST3Gal II did not increase after testosterone treatment in the PC3 cells at any time point up to 120 h (Figure S2). Based on these findings, we hypothesized that the media with castrate levels of testosterone led to the epigenetic silencing of ST3Gal, a gene required for the synthesis of GD1a, in LNCap cells.

### Epigenetic regulation of ST3Gal II in LNCap cells

**Figure 3:**
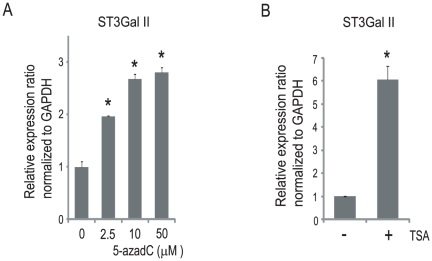
Epigenetic regulation of ST3Gal II in LNCap cells. (A) LNCap cells were treated with 5-aza-2′-deoxycytidine (5-azadC) (0–50 µM) for 120 h, by refeeding with fresh medium with or without 5-azadC at 72 h. The quantitative real-time PCR analyses for ST3Gal II were performed, and the expression levels are reported as the means ± S.E. (n = 3) of the fold difference in mRNA after normalizing the values to the expression level of untreated cells. *P<0.05. (B) LNCap cells were treated with 5 µM trichostatin A (TSA) for 48 h. The quantitative real-time PCR analyses for ST3Gal II were performed, and the expression levels are reported as the means ± S.E. (n = 3) of the fold difference in mRNA after normalizing the values to the expression level of untreated cells. *P<0.05.

Next, we examined whether ST3Gal II was epigenetically regulated in LNCap cells. The LNCap cells were treated with a DNA methyltransferase inhibitor, 5-azadC, and incubated for 120 h (Fig. 3A). The quantitative real-time PCR analyses showed that the expression of ST3Gal II was up-regulated after 5-azadC treatment. Following this experiment, the LNCap cells were treated with a histone deacetylase inhibitor, TSA, and incubated for 48 h (Fig. 3B). The quantitative real-time PCR analyses showed that the expression of ST3Gal II was up-regulated after TSA treatment. These results suggest that epigenetic regulation, including DNA methylation and histone modifications, may be involved in the repression of the ST3Gal II gene in LNCap cells. In further experiments, we focused on DNA methylation at the CpG island in the ST3Gal II promoter.

### Control of DNA methylation at the CpG island in the ST3Gal II gene promoter in prostate cancer cells

**Figure 4:**
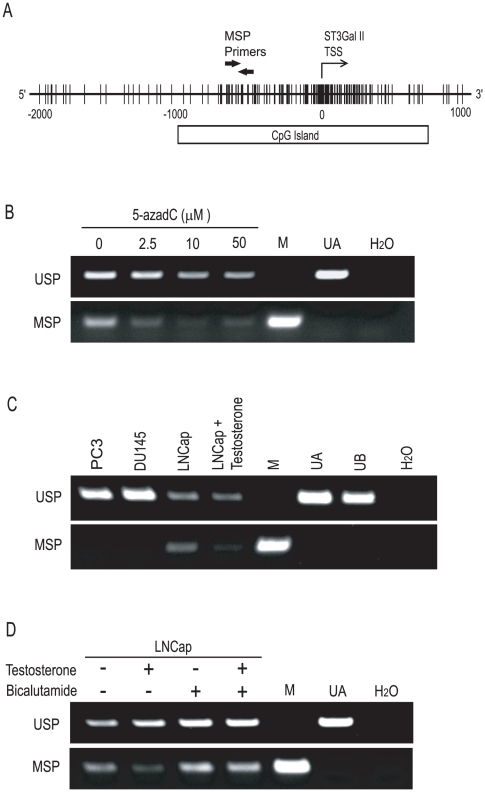
Control of DNA methylation at the CpG island in the ST3Gal II promoter in prostate cancer cells. (A) The CpG island in the ST3Gal II p1 promoter and the location of the MSP primers. The vertical bars represent CpG sites and TSS represents the transcriptional start site. (B–D) The MSP analyses of the CpG island of ST3Gal II. DNA was isolated from LNCap cells treated with 5-azadC (0–50 µM) for 120 h (B), castration-resistant prostate cancer cell lines (PC3 and DU145) or LNCap cells treated with or without 100 nM testosterone for 120 h (C) or LNCap cells treated with or without 100 nM testosterone simultaneously with or without 10 µM bicalutamide for 120 h (D). Then, the DNA was treated with sodium bisulfite, and finally amplified with primers specific for the unmethylated (USP) or the methylated (MSP) form of the CpG island in the ST3Gal II promoter (M, methylated control; UA, unmethylated control A; UB, unmethylated control B). The MSP analyses were repeated 3 times with the same results, and a representative image is shown in the figures.

The gene for human ST3Gal II has been cloned, and the p1 promoter is reportedly necessary for active transcription of this gene in prostate cancer cells [30]. The ST3Gal II promoter sequences are publically available, and we identified a CpG island in the ST3Gal II p1 promoter using the Methyl Primer Express Software program, version 1.0 (Applied Biosystems, Foster City, CA) (Fig. 4A). We examined the methylation at the CpG in the ST3Gal II promoter using the MSP analysis. Genomic DNA was isolated from LNCap cells treated with or without 5-azadC for 120 h, that were then treated with sodium bisulfite, and the DNA was amplified with primers specific for the unmethylated or the methylated ST3Gal II promoter (Fig. 4B). In LNCap cells, the CpG island in the ST3Gal II promoter, which was originally hypermethylated, was demethylated by 5-azadC treatment. Next, we examined the effect of testosterone on the methylation at the CpG in the ST3Gal II promoter in LNCap cells using a MSP analysis (Fig. 4C). In the PC3 and DU145 cells, the CpG island of the ST3Gal II promoter was constitutively hypomethylated. In the LNCap cells, the CpG island of the ST3Gal II promoter was hypermethylated in the absence of testosterone and demethylated in the presence of testosterone. Furthermore, the demethylation at the CpG island in the ST3Gal II promoter after testosterone treatment was suppressed by an anti-androgen, bicalutamide, in LNCap cells (Fig. 4D).

We also examined whether global DNA demethylation is under androgen-dependent control in LNCap cells. We examined the overall restriction patterns of *Msp*I- or *Hpa*II-digested genomic DNA. These enzymes are isoschizomers that recognize the target sequence 5′-CCGG-3′, but the activity of *Hpa*II is inhibited by methylation of the inner cytosine of this sequence. The genomic DNA isolated form LNCap cells treated with or without testosterone was digested using *Msp*I or *Hpa*II (Figure S3A). Testosterone treatment did not greatly affect the digestion pattern of *Hpa*II-treated genomic DNA from LNCap cells, indicating that global DNA demethylation in LNCap cells was not under androgen-dependent control. We then examined the CpG island of GSTP1, which is reported to be hypermethylated during prostate carcinogenesis and also in LNCap cells [27]. Based on the MSP analysis, testosterone treatment did not affect the methylation of GSTP1 in LNCap cells (Figure S3B). Thus, the androgen-dependent control of DNA demethylation may be induced preferentially at the CpG island in the ST3Gal II gene promoter in LNCap cells.

### Androgen-dependent and epigenetic regulation of ST3Gal I in LNCap cells

**Figure 5:**
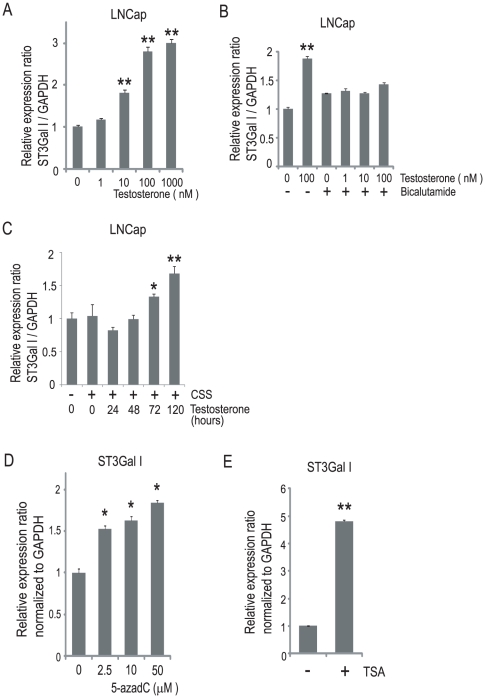
Androgen-dependent and epigenetic regulation of ST3Gal I in LNCap cells. (A) LNCap cells were treated with or without testosterone (0–1000 nM) for 120 h, by refeeding with fresh medium with or without testosterone at 72 h. The quantitative real-time PCR analyses for ST3Gal I were performed, and the expression levels are reported as the means ± S.E. (n = 3) of the fold difference in mRNA after normalizing the values to the expression level of untreated cells. **P<0.001. (B) LNCap cells were treated with or without testosterone (0–100 nM) and simultaneously with or without 10 µM bicalutamide for 120 h, by refeeding with fresh medium with or without testosterone and/or bicalutamide at 72 h. The quantitative real-time PCR analyses for ST3Gal I were performed, and the expression levels are reported as the means ± S.E. (n = 3) of the fold difference in mRNA after normalizing the values to the expression level of untreated cells. **P<0.001. (C) LNCap cells were incubated in charcoal-stripped serum (CSS) for 48 h and then treated with 100 nM testosterone for the indicated times. The quantitative real-time PCR analyses for ST3Gal I were performed, and the expression levels are reported as the means ± S.E. (n = 3) of the fold difference in mRNA after normalizing the values to the expression level of untreated cells. *P<0.05, **P<0.001. (D) LNCap cells were treated with 5-aza-2′-deoxycytidine (5-azadC) (0–50 µM) for 120 h, by refeeding with fresh medium with or without 5-azadC at 72 h. The quantitative real-time PCR analyses for ST3Gal I were performed, and the expression levels are reported as the means ± S.E. (n = 3) of the fold difference in mRNA after normalizing the values to the expression level of untreated cells. *P<0.05. (E) LNCap cells were treated with 5 µM trichostatin A (TSA) for 48 h. The quantitative real-time PCR analyses for ST3Gal I were performed, and the expression levels are reported as the means ± S.E. (n = 3) of the fold difference in mRNA after normalizing the values to the expression level of untreated cells. **P<0.001.

Although GD1a is synthesized from GM1 mainly by ST3Gal II, ST3Gal I may also contribute to the synthesis of GD1a [6], [21]–[24]. We previously reported that ST3Gal I was expressed in LNCap cells, while the expression of ST3Gal II was silenced [20]. Therefore, the regulation of ST3Gal I transcription may be different from that of ST3Gal II. We thereafter examined whether the expression of ST3Gal I, like ST3Gal II, was controlled by testosterone in LNCap cells. In this experiment, the LNCap cells were treated with testosterone and incubated for 120 h. The quantitative real-time PCR analyses showed that testosterone treatment resulted in increased expression of ST3Gal I in LNCap cells (Fig. 5A), although the expression of ST3Gal VI, the sialyltransferase required for the synthesis of sialyl paragloboside, was not induced by testosterone treatment (Figure S4). Furthermore, the testosterone-mediated induction of ST3Gal I in LNCap cells was suppressed by bicalutamide (Fig. 5B). To ensure that there were no androgens present in the cell culture media, LNCap cells were incubated in charcoal-stripped serum for 48 h. The basal level of ST3Gal I was not significantly different between the LNCap cells cultured with 10% FBS and charcoal-stripped serum (Fig. 5C). Then, the LNCap cells were treated with 100 nM testosterone, and the time-course of the changes following testosterone treatment was evaluated. The expression of ST3Gal I increased 72 h after testosterone treatment and remained elevated for more than 120 h in LNCap cells (Fig. 5C). In PC3 and PNT2 cells, no significant increase in the expression of ST3Gal I was detected after testosterone treatment (Figure S5).

Next, we examined whether the regulation of ST3Gal I was epigenetic in LNCap cells, as was the case for ST3Gal II. The LNCap cells were treated with 5-azadC and incubated for 120 h (Fig. 5D). The quantitative real-time PCR analyses showed that the expression of ST3Gal I was up-regulated by 5-azadC treatment. Next, LNCap cells were treated with TSA, and incubated for 48 h (Fig. 5E). The quantitative real-time PCR analyses showed that the expression of ST3Gal I was up-regulated by TSA treatment. Thus, the regulation of ST3Gal I, like the regulation of ST3Gal II, may be epigenetic and androgen-dependent in LNCap cells. It was previously reported that the p1 promoter of the human ST3Gal I gene is necessary for the active transcription of the gene [31]. The ST3Gal I promoter sequences are publically available, and, like ST3Gal II, we identified a CpG island in the ST3Gal I p1 promoter using the Methyl Primer Express Software program, version 1.0 (Applied Biosystems, Foster City, CA) (Figure S6A). The methylation at the CpG island in the ST3Gal I promoter was not detected in LNCap cells or in PC3 or DU145 cells by the MSP analysis (Figure S6B), thus suggesting that the methylation of a genome region other than the CpG island may affect the expression of ST3Gal I in LNCap cells. Thus, the methylation status of the CpG islands which affect the gene expression levels are different between ST3Gal I and II.

### RelB is required for androgen-dependent regulation of ST3Gal I and II in LNCap cells

**Figure 6:**
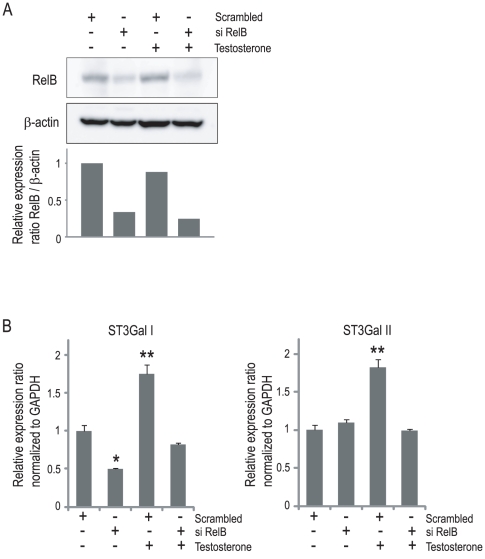
RelB is required for the androgen-dependent regulation of ST3Gal I and II in LNCap cells. (A) LNCap cells were transfected with either scrambled RNA or RelB siRNA and incubated for 120 h with or without 100 nM testosterone. Protein extracts were prepared using RIPA lysis buffer, and the RelB expression level of each sample was analyzed by a Western blot analysis. The expression relative to β-actin is shown in each lane after normalizing the values to the expression level of the scrambled RNA–transfected and testosterone-untreated cells. (B) LNCap cells were transfected with the either scrambled RNA or RelB siRNA and incubated for 120 h with or without 100 nM testosterone. The quantitative real-time PCR analyses for ST3Gal I and II were performed, and the expression levels are reported as the means ± S.E. (n = 3) of the fold difference in mRNA after normalizing the values to the expression level of the scrambled RNA–transfected and testosterone-untreated cells. *P<0.05, **P<0.001.

We next examined whether RelB was required for the testosterone-mediated induction of ST3Gal I and II. LNCap cells were transfected with either scrambled RNA or RelB siRNA and incubated for 120 h. The efficacy of RNAi was assessed by the quantitative real-time PCR analyses (Figure S7). Next, LNCap cells were transfected with either scrambled RNA or RelB siRNA and incubated for 120 h with or without testosterone (Fig. 6). The efficacy of the RelB RNAi was confirmed at the protein level by a Western blot analysis. The levels of RelB protein were not greatly modulated by testosterone treatment (Fig. 6A). The quantitative real-time PCR analyses showed that, without testosterone, RelB siRNA suppressed the expression of ST3Gal I, while no effect was seen on the silenced ST3Gal II. However, the induction of both ST3Gal I and II after testosterone treatment was inhibited by RelB siRNA in the LNCap cells (Fig. 6B). Thus, RelB was required for the androgen-dependent regulation of ST3Gal I and II in LNCap cells.

## Discussion

Gangliosides have been widely investigated because of their relationship with cancer progression [3]. GD1a also appears to be related to cancer cell proliferation and metastasis [11]–[15]. As demonstrated in this study, GD1a was produced in abundance in cancerous tissue samples from human patients with hormone-sensitive prostate cancers as well as those with castration-resistant prostate cancers. However, little is known about the regulation of GD1a production. To address this lack of information, we have been focusing on the transcription of the ST3Gal II gene, which is required for the synthesis of GD1a. We previously reported that ST3Gal II was up-regulated in human castration-resistant prostate cancer cells and that the expression of ST3Gal II is regulated by NF-κB, mainly by RelB [20]. Furthermore, a recent report showed that RelB is activated in human prostate cancers in patients with high Gleason scores [32]. As demonstrated in this study, the expression of ST3Gal II was constitutively activated and androgen-independent in castration-resistant prostate cancer cells (Fig. 2, Figure S2) because the CpG island in the ST3Gal II promoter was hypomethylated (Fig. 4). However, in androgen-depleted LNCap cells, a hormone-sensitive prostate cancer cell line, ST3Gal II was not up-regulated in spite of the activation of RelB.

We herein demonstrated that the expression of the ST3Gal II required for the production of GD1a was epigenetically silenced under androgen-depleted conditions and was up-regulated by androgen-treatment in hormone-sensitive prostate cancer cells. The CpG island of the ST3Gal II promoter was hypermethylated under androgen-depleted conditions and was demethylated by androgen treatment in hormone-sensitive prostate cancer cells (Fig. 4). The presence of the androgen may promote a chromatin environment where RelB can activate the transcription of ST3Gal II in hormone-sensitive prostate cancer cells. Thus, in hormone-sensitive prostate cancers, the production of GD1a may be regulated by androgen, which can modulate the methylation state of the CpG sites in the promoter region of a sialyltransferase gene.

Although effects of androgen on NF-κB activity have been reported, the topic remains controversial. One report showed that androgen treatment repressed the NF-κB activity through maintenance of the IκBα protein levels [33], but another report showed that the NF-κB DNA binding activity increased after androgen treatment [34]. In the present manuscript, the NF-κB activity was not significantly different between the testosterone-treated LNCap cells compared to the cells cultured without testosterone (Figure S1). The two routes in NF-κB signaling are the canonical pathway, which involves the complex formed between RelA and p50, and the non-canonical pathway, in which the complex formed between RelB and p52 is involved [35]. The different subunits of NF-κB may be differently regulated by androgens. Although the levels of nuclear RelA and p52 were not elevated after androgen treatment in a previous report [34], it is still unknown whether androgens can modulate the level of RelB. In the present study, we showed that the levels of RelB protein were not greatly modulated by androgens in the cell lines examined (Fig. 6).

Although GD1a is synthesized from GM1 mainly by ST3Gal II, ST3Gal I may also contribute to the synthesis of GD1a [6], [21]–[24]. We found that the expression of ST3Gal I was up-regulated by 5-azadC treatment in LNCap cells, indicating that the expression of ST3Gal I may be regulated by DNA methylation, as is the case for ST3Gal II. However, methylation at the CpG island in the ST3Gal I promoter was not detected in LNCap cells by a MSP analysis. This suggests that the methylation of a genome region other than the CpG island may affect the expression of ST3Gal I. Thus, the methylation status of CpG islands which affect the gene expression are different between ST3Gal I and II. As previously reported [20], in LNCap cells, ST3Gal I was expressed, while ST3Gal II expression was silenced. The differences in the methylation of the CpG island between ST3Gal I and II may result in the differences in the expression of these two genes in LNCap cells. Based on publically available data and previous studies [36], [37], we identified that ST3Gal II has one highly probable androgen receptor recruitment site −39.5 kb of the transcription start site of the ST3Gal II p1 promoter, and that the ST3Gal I has two highly probable androgen receptor recruitment sites −13.9 kb and −42.6 kb of the transcription start site of the ST3Gal I p1 promoter. Thus, as there are differences in the methylation status of CpG islands, the regulation of promoter methylation by the androgen receptor might also be different between ST3Gal I and II.

The progression of many cancers is epigenetically regulated. DNA demethylation changes have been reported to occur later in prostate carcinogenesis [38], [39]. We first demonstrated that the androgen-dependent activation of transcription was induced by demethylation of CpG promoter region in ST3Gal II. Recently, several reports have shown that DNA methylation can be controlled by hormone receptors. For example, the estrogen receptor directly modifies the methylation status of the pS2 gene [40], [41], and the glucocorticoid receptor could also modify the DNA methylation status [42]. Another report showed that DNA methylation/demethylation was hormonally altered to control the transcription of the cytochrome p450 27B1 gene, and that the 5-methyl-CpG binding domain family (MBD) protein activated by hormonal stimulation seemed to complete the DNA demethylation in the MBD-bound promoter [43]. In the present study, we demonstrated that demethylation of the ST3Gal II promoter was induced by androgen treatment in hormone-sensitive prostate cancer cells. Although the mechanism is currently unclear, the MBD protein may be involved in this type of androgen-induced DNA demethylation. Further research is needed to elucidate the mechanism underlying the hormonal control of the DNA methylation/demethylation of the ST3Gal II promoter.

Androgen plays a pivotal role in the development, growth, and progression of prostate cancers [25]. Although we have herein shown the androgen-dependent activation of ST3Gal II by the demethylation of CpG promoter region, other genes may also be epigenetically regulated by androgen treatment in hormone-sensitive prostate cancer cells. Although we focused on DNA methylation at the CpG island in the ST3Gal promoter in the current manuscript, it is known that DNA methylation is linked to histone deacetylation [44], [45]. Therefore, in future studies, we plan to elucidate the mechanism underlying the regulation on histone modification by androgen, in addition to the effects on DNA methylation.

GD1a should be focused also from the view of cancer therapy. Several gene therapy approaches for the treatment of prostate cancer have been clinically tested [46]. Oncolytic viruses have been developed to selectively augment the anti-tumor effects, and some viruses, such as adenovirus and the herpes simplex virus, are used to combat prostate cancers [47], [48]. Recently, we reported that inactivated Sendai virus particles (HVJ-E) selectively induced apoptosis in human castration-resistant prostate cancer cells by retinoic acid-inducible gene-I (RIG-I)–mediated gene expression and the induction of anti-tumor immunities [17]. A ganglioside, GD1a, which is enriched in human castration-resistant prostate cancer cells [17]–[20] is one of the receptors for the Sendai virus [16]; therefore, HVJ-E is expected to be a novel therapeutic tool for prostate cancers. However, HVJ-E did not induce apoptosis in LNCap cells, a human hormone-sensitive prostate cancer cell line, because these cells did not express a viral receptor ganglioside, such as GD1a [17], on their cell surface. The present study showed that GD1a was produced in clinical samples of hormone-sensitive prostate cancers and of castration-resistant prostate cancers. We are currently analyzing the mechanism underlying the cancer-selective apoptosis induced by HVJ-E, and our preliminary data suggest that both castration-resistant and hormone-sensitive prostate cancers may be treated by HVJ-E. Thus, this study will also be important to determine the indications for treating prostate cancer patients with HVJ-E.

## Supporting Information

Figure S1 **NF-κB activity after testosterone treatment in LNCap cells.** LNCap cells were transfected with a NF-κB luciferase reporter construct and incubated for 120 h with or without 100 nM testosterone. The luciferase activity was measured, and the results are shown as the means ± S.E. (n = 3).
(EPS)

Figure S2 **Androgen-independent regulation of ST3Gal II in PC3 cells.** PC3 cells were incubated in charcoal-stripped serum (CSS) for 48 h and then treated with 100 nM testosterone for the indicated times. The quantitative real-time PCR analyses for ST3Gal II were performed, and the expression levels are reported as the means ± S.E. (n = 3) of the fold difference in mRNA after normalizing the values to the expression level of untreated cells.
(EPS)

Figure S3 **Testosterone does not induce global DNA demethylation in LNCap cells.** (A) The results of the differential restriction analysis. The genomic DNA isolated from untreated LNCap cells or LNCap cells treated with 100 nM testosterone for 120 h was digested using *Msp*I or *Hpa*II for 16 h at 37°C. The digested DNA was analyzed in 2% agarose gels stained with ethidium bromide. (B) The results of the MSP analysis of GSTP1. The genomic DNA isolated from untreated LNCap cells or LNCap cells treated with 100 nM testosterone for 120 h was amplified with primers specific for the unmethylated (USP) or the methylated (MSP) GSTP1 promoter after treatment with sodium bisulfite (M, methylated control; UA, unmethylated control A; UB, unmethylated control B). The MSP analyses were repeated 3 times with the same result and a representative image shown in the figure.
(EPS)

Figure S4 **Androgen-independent regulation of ST3Gal VI in LNCap cells.** LNCap cells were treated with testosterone (0–1000 nM) for 120 h, by refeeding with fresh medium with or without testosterone at 72 h. The quantitative real-time PCR analyses for ST3Gal VI were performed, and the expression levels are reported as the means ± S.E. (n = 3) of the fold difference in mRNA after normalizing the values to the expression level of untreated cells.
(EPS)

Figure S5 **Androgen-independent regulation of ST3Gal I in PC3 and PNT2 cells.** PC3 and PNT2 cells were treated with testosterone (0–1000 nM) for 120 h, by refeeding with fresh medium with or without testosterone at 72 h. The quantitative real-time PCR analyses for ST3Gal I were performed, and the expression levels are reported as the means ± S.E. (n = 3) of the fold difference in mRNA after normalizing the values to the expression level of untreated cells.
(EPS)

Figure S6 **Control of DNA methylation at the CpG island in the ST3Gal I promoter in prostate cancer cells.** (A) The CpG island in the ST3Gal I p1 promoter and the location of the MSP primers. The vertical bars represent CpG sites and TSS represents the transcriptional start site. (B) The MSP analysis of ST3Gal I. DNA was isolated from castration-resistant prostate cancer cell lines (PC3 and DU145) or LNCap cells which were treated with or without 100 nM testosterone for 120 h, and then treated with sodium bisulfite, and was finally amplified with primers specific for the unmethylated (USP) or the methylated (MSP) form of the CpG island in the ST3Gal I promoter (M, methylated control; UA, unmethylated control A; UB, unmethylated control B). The MSP analyses were repeated 3 times with the same result and a representative image shown in the figure.
(EPS)

Figure S7 **The efficacy of RelB RNAi as assessed by the quantitative real-time PCR analyses.** LNCap cells were transfected with either scrambled RNA or RelB siRNA and incubated for 120 h. The total RNA from LNCap cells transfected with either the scrambled RNA or RelB siRNA were subjected to the quantitative real-time PCR analyses, and the results are shown as the means ± S.E. (n = 3). **P<0.001.
(EPS)
